# Supplementary material for: Evaluating Observer Reliability and Diagnostic Accuracy of CT-LEFAT Criteria for Post-Treatment Head and Neck Lymphedema: A Prospective Blinded Comparative Analysis of Oncologist Human Inter-Rater Performance
Source: medRxiv. 2024 Sep 18:2024.09.17.24313809. Preprint. [Version 1] doi: 10.1101/2024.09.17.24313809 (PMC11451822; doi:10.1101/2024.09.17.24313809)
Supplement: Supplement 2 [file media-2.pdf]

#	Variable / Field Name	Field Label<br>*Field Note*	Field Attributes (Field Type, Validation, Choices, Calculations, etc.)
**Instrument: Subject ID (subject_id)**			
1	[subject_id]	Subject ID	text, Identifier
2	[subject_last_name]	Last Name	text
3	[subject_id_complete]	Section Header: *Form Status*<br>Complete?	dropdown<br>0 Incomplete<br>1 Unverified<br>2 Complete
**Instrument: Reader (reader)**			
4	[description]	The objective of this form is to validate the evaluation criteria developed for assessing radiation-induced changes (CT-LEFAT) as outlined in the original publication by Aulino et al	descriptive
5	[img_modality_lefat]	Please indicate the modality used for the assessment<br>*Please select image modality/protocol you used for the evaluation*	dropdown<br>1 CECT (W/L 350/40 HU)<br>2 MRI T2 FS<br>3 MRI T1+/-C
6	[study_date_lefat]	Imaging Study Date<br>*Please enter study date YYYY-MM-DD*	text (date_ymd)<br>Field Annotation: @HIDEBUTTON
7	[reader_name_lefat]	Reader's Name<br>*Please enter your first and last name. You are logged in as [user-fullname]*	text, Required, Identifier<br>Field Annotation: @DEFAULT="[user-fullname]"
8	[reader_initials_lefat]	Reader's Initials	text, Required, Identifier<br>Field Annotation: @USERNAME
9	[description_fat_stranding]	Fat Stranding Evaluation:Please evaluate and grade the presence and extent of fat stranding in the following locations on a scale of 0-2 where 0-normal, 1-mild changes, and 2-advanced. If a location is not evaluable, please select N/A. Location Laterality Evaluation of Fat Stranding Presence and Extent Anterior Cervical Space Axial (at the superior thyroid cartilage) Right {ant_cerv_r} Left {ant_cerv_l} Posterior cervical spaces Axial (at the superior thyroid cartilage) Right {post_cerv_r} Left {post_cerv_l} Anterior neck subcutaneous fat Axial (at the superior thyroid cartilage) Midline {ant_scf} Submental region Sagittal (superficial and deep to the platysma muscle) Midline {submental}	descriptive
10	[ant_cerv_r]	Anterior cervical space (Right)	radio (Matrix), Required<br>0 0<br>1 1<br>2 2<br>3 N/A
11	[ant_cerv_l]	Anterior cervical space (Left)	radio (Matrix), Required<br>0 0<br>1 1<br>2 2<br>3 N/A
12	[post_cerv_r]	Posterior cervical space (Right)	radio (Matrix), Required<br>0 0<br>1 1<br>2 2<br>3 N/A
13	[post_cerv_l]	Posterior cervical space (Left)	radio (Matrix), Required<br>0 0<br>1 1<br>2 2<br>3 N/A
14	[ant_scf]	Anterior neck subcutaneous fat (midline)	radio (Matrix), Required<br>0 0<br>1 1<br>2 2<br>3 N/A
15	[submental]	Midline sagittal submental region	radio (Matrix), Required<br>0 0<br>1 1<br>2 2<br>3 N/A
16	[submucosa_edema]	Submucosal edema evaluation: Please evaluate the volume changes using diameter of the following structures: AP diameter of the epiglottis, calculated 8 mm from the free margin (superior extent) to the anterior surface of the epiglottis, and then to the closest point on the posterior surface. Epiglottis Diameter (in mm): {epigl_thickness} Prevertebral soft tissue (PVST) at the mid-C3 level PVST Diameter (in mm): {pvst_thickness}	descriptive
17	[epigl_thickness]	Please enter the epiglottis thickness in millimeters	text (number)<br>Custom alignment: RH
18	[pvst_thickness]	Please enter the prevertebral soft tissue thickness (PVST) at the level of mid-C-3 in millimeters	text (number)<br>Custom alignment: RH
19	[eval_date_lefat]	Evaluation Date	text (date_ymd)
